# Evolution of talent policies in Guangdong-Hong Kong-Macao greater bay area: An LDA thematic model approach

**DOI:** 10.1371/journal.pone.0336580

**Published:** 2025-12-01

**Authors:** Jinpeng Wen, Hongxing Han

**Affiliations:** School of Journalism and Communication, South China University of Technology, Guangzhou, China; Macau University of Science and Technology, MACAO

## Abstract

As construction of the Guangdong-Hong Kong-Macao Greater Bay Area advances, talent has become the core driving force for its development. This study quantitatively analyzes 94 talent policies from Guangdong, Hong Kong, and Macao, revealing the strategic focuses and thematic evolution of these policies across different stages from a time-series perspective. It also distills relevant experiences to propose optimization strategies and a development blueprint for talent policies in the region. By combining historical review and thematic modeling, this study categorizes talent policies from the three regions over the past five years (2019–2024) into three phases: initial phase, developmental phase, and deep plowing phase. Theme heat analysis and similarity analysis are then applied to clarify the strategic focus and evolutionary trajectory of each phase, while the implementation effects of the talent policies are evaluated based on four dimensions: talent inflow volume, talent satisfaction, economic impact, and social influence. The Latent Dirichlet Allocation (LDA) model uncovers seven strategic talent themes and their heat distribution across the three phases. The study reveals that “Hong Kong and Macao tax incentives” have consistently served as the cornerstone of talent policy throughout the development process, while also fostering the rapid emergence of related themes. Looking ahead, efforts will be dedicated to building a comprehensive talent ecosystem integrating education, science and technology, and social security, thereby positioning the Guangdong-Hong Kong-Macao Greater Bay Area as an international first-class bay area. Finally, this paper draws on the successful experiences of the Yangtze River Delta, Beijing-Tianjin-Hebei region, and other international bay areas to formulate targeted strategies for the development of the Guangdong-Hong Kong-Macao Greater Bay Area.

## 1. Introduction

Xi Jinping emphasized that talent, as the foremost resource for development, should be leveraged to deeply implement the talent-strong nation strategy in the new era, aiming to establish a hub of high-end talent in Beijing, Shanghai, and the Guangdong-Hong Kong-Macao Greater Bay Area to support the modernization goal by 2035 [1]. The Bay Area Economy, a highly significant form of development, is increasingly recognized for its importance on the global stage and its pivotal role in advancing economic globalization. World Bank research indicates that approximately 60% of the global economic output is derived from ports, bay areas, and their surrounding inland regions. The Global Cities Index Report 2023 by Schroders reveals that most of the top 30 global urban areas are situated in bay regions. In March 2015, China initially proposed the establishment of the Guangdong-Hong Kong-Macao Greater Bay Area, which was subsequently integrated into the national development strategy in 2018. In 2023, the Guangdong Provincial Bureau of Statistics published the “Guangdong-Hong Kong-Macao Greater Bay Area Joint Statistical Handbook 2023”, reporting that the region’s total economic output surpasses 13 trillion yuan, with a per capita GDP that ranks second globally among bay areas. Typical examples of premier international bay areas include the New York Bay Area, San Francisco Bay Area, and Tokyo Bay Area, all of which are globally recognized as financial, service, shipping, and innovation centers [2]. Empirical evidence suggests that international bay areas have emerged as the principal catalysts for global economic development and are at the forefront of technological innovation. Yet, when juxtaposed with the Beijing-Tianjin-Hebei region, the Yangtze River Delta, and international bay areas, the Guangdong-Hong Kong-Macao Greater Bay Area confronts issues such as talent distribution disparities [3], suboptimal talent structure [4], and deficiencies in talent attraction and incentive mechanisms [5]. Knowledge-laden talent is increasingly becoming a primary source of economic growth, with the silent battle for talent unfolding both internationally and regionally, where ownership of talent equates to a competitive edge. Against this backdrop, examining the talent policy trends in the Guangdong-Hong Kong-Macao Greater Bay Area over the years offers crucial insights into the evolution of pertinent issues and holds substantial significance for China’s future industrial and economic trajectory. Therefore, this paper proposes the following four research questions:

RQ1:What characteristics have defined the evolution of the talent policy in the Guangdong-Hong Kong-Macao Greater Bay Area over the past five years?

RQ2:What have been the key themes within the talent policy framework over the past five years?

RQ3:How has the thematic focus evolved across different stages?

RQ4:How are the themes at various stages interconnected with the policy’s evolution?

RQ5:How effective is the implementation of the Guangdong-Hong Kong-Macao Greater Bay Area talent policy?

However, Recent research on talent development within the Guangdong-Hong Kong-Macao Greater Bay Area has predominantly centered on analyzing the current state and talent demands [6], policy and system development [7], talent aggregation, innovation, and entrepreneurship [8], evaluation and incentive mechanisms [9], as well as education and cultivation [10]. In studying the talent policies of the Guangdong-Hong Kong-Macao Greater Bay Area, the research approach mainly relies on policy documents from different years. For example, bibliometric methods have been used to quantitatively analyze the government’s historical focus on talent development [11]. Comparative content analysis has been employed to identify differences and commonalities in talent policies across regions, and empirical research has been conducted to assess the actual effectiveness of these policies [12,13]. These methods complement each other, collectively revealing the government’s focus, implementation level, and emphasis on talent development over the years. This, in turn, provides strong support for identifying current issues and proposing optimization strategies. This approach aims to delineate the current state of affairs and propose optimization strategies. However, the research paradigm for such policy texts remains relatively traditional, primarily conducting macroscopic analyses with a focus on overall packaging of the text, neglecting to explore the interannual correlations within policy themes. To address these limitations, this study employs Potential Delicacy’s LDA model for a comprehensive analysis of the development and evolution of talent policies in the Guangdong-Hong Kong-Macao Greater Bay Area from 2019 to 2023. Initially, this study systematically catalogues the implementation of 94 talent policies in the Guangdong-Hong Kong-Macao Greater Bay Area and summarizes their maturation processes. Subsequently, following data preprocessing, the LDA model extracts keyword features from the policy texts and analyzes the distribution and content of potential themes. Building on this foundation, the data is further decomposed by temporal windows to investigate the evolution of theme intensity and content chronologically. The ultimate objective is to elucidate the intricate interplay between talent policies in Guangdong-Hong Kong-Macao and their developmental trajectories, offering theoretical insights for regional talent strategic planning.

Compared to previous studies, the innovations presented in this paper include: (1) The research methodology employed is innovative. For the talent policy of the Guangdong-Hong Kong-Macao Greater Bay Area over the past five years, an innovative approach has been utilized, leveraging Python technology to extract and preprocess data text. Subsequently, the LDA model is applied to discern thematic progressions, assess theme similarities, and generate heat maps and Sankey diagrams. This methodology unveils the evolution trajectories and challenges of talent development themes and draws on the experiences of other bay areas to propose developmental recommendations. (2) The selection of a highly timely topic. Marking the over six-year implementation of the Outline Development Plan for the Guangdong-Hong Kong-Macao Greater Bay Area (hereinafter referred to as the “Outline Plan”), which was jointly issued by the Central Committee of the Communist Party of China and the State Council in 2019, this study addresses a pivotal juncture for reviewing national outcomes and future planning, thereby bridging a significant gap in the research on talent policy during that period.

## 2. Literature review

### 2.1 Study on talent policies in the bay area

The Bay Area Talent Policy consistently remains a topic of keen interest among scholars internationally. Research on the Bay Area Talent Policy across the globe encompasses various disciplines, including sociology, public administration, education, and economics, all of which persistently concentrate on the development of talent within the Bay Area. Policies within the Bay Area that center on talent mobility [14], strategic planning [15], industry-education integration [16], incentives [17], housing [18], scientific and technological talent reserves [19], and immigration [20] are all highly debated topics. Notably, the migration of talent within the Bay Area is frequently complemented by the movement of technology, capital, information, and other elements, forming a critical foundation for regional competitiveness. Amid today’s accelerating globalization and regional integration, talent emerges as a core driver of technological innovation and regional development. Major global Bay Areas—New York, San Francisco, Tokyo, and the Guangdong-Hong Kong-Macao Greater Bay Area—are actively constructing unique talent ecosystems to attract and retain top talent, thereby enhancing regional competitiveness. The New York Bay Area, with its open economic structure and efficient resource allocation, serves as the capital of international migrants. Its multicultural environment offers ample development opportunities for talents. The San Francisco Bay Area has developed a unique science and innovation ecosystem. By leveraging the digital industrialization of information technology and biotechnology, it has adopted a self-organized development model that stimulates talent innovation. The Tokyo Bay Area has concentrated numerous high-quality talents through government intervention and market coordination. It ranks among the most mobile regions within the Asia-Pacific [21]. Conversely, the Guangdong-Hong Kong-Macao Greater Bay Area, a vital economic growth pole in China, has achieved significant advancements in its talent policies recently. By implementing preferential policies—such as personal income tax exemptions, housing subsidies, and research start-up funds—the Guangdong-Hong Kong-Macao Greater Bay Area has successfully attracted numerous high-end talents and innovative resources. Nevertheless, the Guangdong-Hong Kong-Macao Greater Bay Area lags behind top international Bay Areas in talent concentration and appeal. For instance, San Francisco Bay Area’s labor force with bachelor’s degrees or above constitutes 46%, versus the Guangdong-Hong Kong-Macao Greater Bay Area’s 17.47%. This highlights the need for improvement in the Guangdong-Hong Kong-Macao Greater Bay Area’s talent cultivation and attraction [22]. Recent studies reveal a new trend in global talent mobility. China is becoming a magnet for global talent, with the Guangdong-Hong Kong-Macao Greater Bay Area demonstrating strong talent attraction attributed to its large economic volume and complete industrial chain. The 2023 data indicates that the Guangdong-Hong Kong-Macao Greater Bay Area’s technology clusters rank among the world’s top innovators, attracting more “high-precision and sharp-shortage” talents. Concurrently, global talent mobility is shifting from permanent relocation to flexible attraction. This shift offers new opportunities for the Guangdong-Hong Kong-Macao Greater Bay Area to utilize digital platforms and private enterprises, creating a global talent oasis [23]. To further enhance the Guangdong-Hong Kong-Macao Greater Bay Area’s talent competitiveness, future policies should emphasize regional cooperation, resource allocation optimization, and synergistic industrial development. By enhancing the talent policy system, increasing policy precision and effectiveness, and constructing an ecosystem to boost talent innovation and vitality, the Guangdong-Hong Kong-Macao Greater Bay Area is anticipated to gain a more advantageous position in the global talent competition, offering robust intellectual support for economic prosperity and social progress.

It is worth noting that Chinese scholars has been proactive in developing talent policies within the Guangdong-Hong Kong-Macao Greater Bay Area, where Hong Kong and Macao emphasize environmental services and welfare protection, while Guangzhou and Shenzhen broadly address talent attraction, development, and incentives. Recently, the Greater Bay Area has implemented several key talent policies, including Hong Kong’s “Quality Migrant Admission Scheme”, Guangzhou’s “Cotton Tree Program”, and Shenzhen’s “Peacock Program”, all designed to attract and nurture high-caliber talent. It merits attention that scholarly research on talent policy in the Bay Area predominantly concentrates on talent introduction [24], collaborative talent development [25], scientific and technological innovation [11], and comparative analyses between regions such as the Beijing-Tianjin-Hebei, Yangtze River Delta, and Guangdong-Hong Kong-Macao Bay Areas [26]. As Internet technology advances and interdisciplinary research methods evolve, policy text research has progressed from rudimentary summarization of initial experiences and international references to a diverse array of methodological approaches, including bibliometric analysis, comparative studies, empirical analysis, surveys, hierarchical analysis, and expert consultation, with the depth of research content and the validity of indices enhancing through ongoing academic innovation.Text mining, LDA modeling, thematic clustering, and network semantic analysis are among the methodologies that have been progressively integrated into the study of talent policy texts.

### 2.2 Policy text theme mining

To address the need for a critical evaluation of text-mining methods in policy analysis, we present a structured comparison in Table 1 to clarify the strengths, weaknesses, and applicability of mainstream methods within the context of policy research. Policy text theme mining decodes the thematic structure, policy orientation, and strategic logic of policy documents via text analysis techniques [27]. For policy analysis scenarios involving complexities such as cross-domain differences and multi-stage evolution, a critical comparison of these methods is essential to justify methodological choices.

**Table 1:** Method comparison [28,29].

Method	Core Principle	Strengths in Policy Analysis	Weaknesses in Policy Analysis	Applicability to This Study
**TF-IDF**	Evaluate keyword importance via term frequency (TF) and inverse document frequency (IDF)	1. High interpretability: Quickly identifies core keywords. 2. Low computational complexity	1. Ignores semantic connections between keywords. 2. Overemphasizes generic terms that mask distinctive themes	Auxiliary: Verifies thematic consistency; not for latent relationship mining
**LDA**	Documents = theme mixtures; Themes = keyword mixtures (Bayesian framework)	1. Uncovers latent thematic links across documents. 2. Tracks theme evolution over time	1. Needs parameter (α/β) tuning; poor settings lead to unfocused themes. 2. Technical concepts may confuse non-technical readers	Core: Addresses exploration of latent themes and their evolution
Co-word Analysis	Constructs keyword co-occurrence networks to identify clusters	1. Visualizes explicit keyword connections. 2. Easy to replicate with minimal parameters	1. Misses implicit semantic connections between keywords. 2. Fails with sparse keyword distributions	Not adopted: Unsuitable for latent theme and evolution analysis
**Supervised Learning (e.g., SVM)**	Classifies texts via manually labeled training data	1. High accuracy for predefined themes (e.g., “policy type A” vs. “policy type B”). 2. Predicts themes of new policy documents	1. Requires heavy manual labeling (inefficient for large-scale policy corpora). 2. Cannot detect emerging themes not included in initial labels	Not adopted: Inflexible for exploring unanticipated latent themes in evolving policies

As shown in Table 1, TF-IDF excels in interpretability (quickly identifying core keywords) but ignores semantic connections (failing to link semantically related terms to the same theme), making it suitable only as an auxiliary tool for preliminary keyword extraction. In contrast, LDA, by modeling documents as mixtures of latent themes, uncovers critical latent thematic links and tracks theme evolution over time, positioning it as a core method for exploring hidden thematic structures and their dynamic changes. Co-word Analysis, while useful for visualizing explicit keyword co-occurrence links, misses implicit policy logics and struggles with sparse data, thus limiting its applicability in-depth policy thematic analysis. This comparative evaluation ensures methodological choices are grounded in the unique demands of policy text mining.

### 2.3 Policy evolution analysis studies

The exploration of policy evolution not only reflects the industry’s historical experiences and strategic developmental concepts but also elucidates governmental policymaking inclinations and forecasts developmental trends, thereby offering robust support for policy analysis and judgment [30,31]. Scholars typically employ historical analysis when examining policy evolution, a methodology that emphasizes the systematic collation and interpretation of policy development through pivotal events, milestone occurrences, and pertinent documentation [32]. As an illustration, China’s strategic scientific and technological policy development has been compartmentalized into three distinct phases—initial exploration, strategic enhancement, and comprehensive development—each exhibiting unique characteristics [33]. Within the realm of quantitative research, scholars frequently employ econometrics in conjunction with co-occurrence analysis, social network analysis, text mining, and other techniques for exploration. Recently, an escalation in the quantity of research outcomes utilizing quantitative methodologies to outline the stages of policy evolution has been observed. Wu Ruipeng et al. initially conducted a systematic organization of the U.S. AI strategy’s development and delineated distinct temporal phases. Thereafter, a DTM model was utilized to ascertain the prevalence of policy themes, and through the integration of similarity calculations, the thematic evolution of the U.S. AI strategy was deeply examined using visualization techniques such as heat maps and Sankey diagrams. This research not only uncovers the central themes of AI strategy across various historical periods but also provides a detailed analysis of its evolutionary path and the strategic rationale underpinning it [34]. In light of these findings, this study employs a hybrid approach of historical review and thematic modeling to systematically outline the developmental stages of talent policies in the Guangdong-Hong Kong-Macao Greater Bay Area, probing the gradual evolution and articulating the intrinsic logic and principal impetus for policy shifts.

## 3. Study design

### 3.1 Research framework

**Fig 1:**
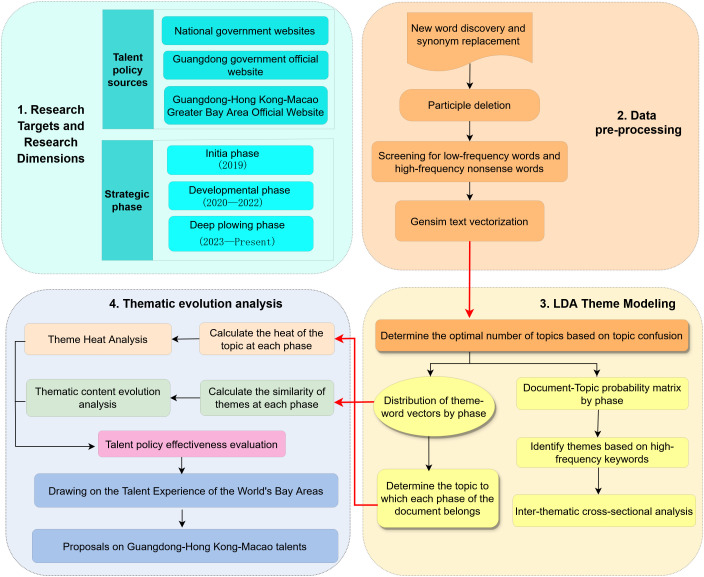
Research framework.

The research roadmap of this paper, as depicted in Fig 1, is designed to employ the LDA topic model for an in-depth analysis of talent policies within the Guangdong-Hong Kong-Macao Greater Bay Area. The primary research steps are outlined below: (1) A crawler gathers talent policy documents from the Greater Bay Area, organizing them based on chronological order and content themes; (2) Data preprocessing involves tokenization, removing discontinuities, and vectorization; (3) The number of topics is determined based on perplexity scores, followed by horizontal analysis; (4) Calculating the heat of the themes to ascertain the focal points of talent strategy at various stages; (5) Conducting theme association analysis to elucidate the progression of strategic themes across stages; (6) Talent policy effectiveness evaluation; (7) Leveraging the successful experiences of the Beijing-Tianjin-Hebei region, the Yangtze River Delta, and global bay areas to propose development strategies for the Guangdong-Hong Kong-Macao Greater Bay Area.

### 3.2 Research methodology

#### 3.2.1 LDA topic model.

The Latent Dirichlet Allocation (LDA) model, proposed by Blei et al., is a Bayesian hierarchical model widely used in text mining [35]. To help non-technical readers understand its core logic, we can use a simple analogy: if each talent policy document is a “fruit salad,” then each “fruit” in the salad represents a keyword (e.g., “tax incentive,” “housing subsidy”), and each “flavor of salad” corresponds to a policy theme (e.g., “Hong Kong and Macao Tax Incentives,” “Young Talent Development”). The LDA model’s role is to “taste” all 94 “salads” (policy documents) and automatically identify how many unique “flavors” (themes) exist, as well as which “fruits” (keywords) make up each “flavor” and how much of each “flavor” is present in each “salad.”

In terms of model structure, the LDA model has a three-tier hierarchy (document-topic-vocabulary) and uses two key parameters—α (alpha) and β (beta)—to adjust the distribution of themes and keywords [36,37]. We can explain these parameters through practical policy scenarios:α(document-topic distribution hyperparameter): Imagine a policy document focusing on “talent training.” If α is set too high (e.g., α = 1.0), the document might be mixed with irrelevant themes like “financial talent promotion,” making the theme unfocused [38]. After multiple experiments, we set α = 0.1, which ensures each policy document mainly centers on 1–2 core themes (e.g., a document about “postdoctoral training” will not be diluted by unrelated content like “lawyer settlement”), aligning with the targeted nature of government talent policies.β (topic-vocabulary distribution hyperparameter): For the theme “Hong Kong and Macao Tax Incentives,” if β is too large (e.g., β = 0.1), the theme might include keywords like “vocational training” that are unrelated to taxation, leading to ambiguous theme connotations. We set β = 0.01, which ensures each theme is composed of highly relevant keywords (e.g., “personal income tax,” “Hengqin Cooperation Zone,” “tax subsidy” for the taxation theme), making it easy for readers to quickly grasp the core of each theme [39].

This parameter configuration (α = 0.1, β = 0.01) not only avoids the problem of “overfitting” (where the model only fits the sample data but fails to generalize to other policy texts) but also ensures the identified themes match the actual policy orientation of the Guangdong-Hong Kong-Macao Greater Bay Area. Compared with the TF-IDF method (which only extracts high-frequency keywords like “talent” and “tax” without revealing thematic connections), the LDA model can dig out hidden logical relationships between policies—for example, how “Hong Kong and Macao tax incentives” drive the development of “lawyer settlement” and “young talent employment” themes.

#### 3.2.2 Comparison of multiple text mining methods.

To further clarify the advantages of LDA in this study, we supplement the following critical comparison with a focus on policy analysis scenarios: In terms of thematic granularity, TF-IDF extracts isolated keywords (e.g., “tax”, “housing”) that cannot distinguish between “Hong Kong-Macao tax incentives” and “mainland housing subsidies”—a critical distinction for cross-jurisdictional policy analysis [40]—whereas LDA groups semantically related keywords into distinct themes, enabling precise comparison of policy focuses across Guangdong, Hong Kong, and Macao [41]; regarding adaptability to policy evolution, Co-word analysis can only show static keyword networks (e.g., 2019 vs. 2023) but cannot explain why themes change (e.g., why “high-precision talent” replaced “basic skill training” as a focus), while LDA’s theme intensity tracking (via heat maps) and evolutionary path analysis (via Sankey diagrams) directly address this gap, supporting the study’s analysis of policy evolution across three phases (RQ3); in terms of practical policy implications, supervised learning requires predefined themes, which limits its ability to inform policy optimization—for example, it cannot identify unmet needs (e.g., the lack of “cross-border pension mutual recognition” in existing policies)—whereas LDA’s latent theme mining reveals such gaps, providing actionable insights for policy refinement [42].

## 4. Data sources and pre-processing

### 4.1 Text acquisition and data pre-processing

We collected 94 talent policy documents (2019–2024) from official platforms—including the National Government Website, Guangdong Provincial Government Portal, and Guangdong-Hong Kong-Macao Greater Bay Area Official Website (a selection of which is displayed in Table 2)—using Python’s requests (v2.31.0) and BeautifulSoup4 (v4.12.2) libraries; the crawler code was configured to avoid duplicate downloads by verifying document MD5 values, and unstructured text (e.g., tables, appendices) was converted to plain text via python-docx (v0.8.11) to ensure consistency across the dataset. For data preprocessing, we first performed word segmentation using the jieba library (v0.42.1) with a custom dictionary that included policy-specific terms such as “Guangdong-Hong Kong-Macao Greater Bay Area” and “Peacock Program”, which prevented incorrect segmentation of professional terminology; next, we removed generic stopwords (e.g., “the”, “at”) using the scikit-learn (v1.3.0) stop_words list, along with policy-specific trivial terms (e.g., “Notice”, “Measures”) identified through manual screening; finally, we converted the preprocessed text into a document-term matrix using the Dictionary and Corpus functions from the gensim library (v4.3.2), filtering out words with a document frequency of less than 3 to reduce data noise.

**Table 2:** Selected Guangdong, Hong Kong and Macao Talent Policies.

	Title of policy report	Publishing time	publishing organization
**National government websites**	Outline Development Plan for the Guangdong-Hong Kong-Macao Greater Bay Area	2019	State Council (PRC)
	Notice on Preferential Policies on Individual Income Tax in Guangdong, Hong Kong and Macao Greater Bay Area	2020	PRC tax authority
	Overall Plan for the Construction of Hengqin Guangdong-Macao Deep Co-operation Zone	2020	State Council (PRC)
	Circular on the Overall Scheme on Deepening World-oriented Guangdong-Hong Kong-Macao Comprehensive Co-operation in Guangzhou Nansha	2022	State Council (PRC)
**Guangdong government official website**	Notice on Continuing the Work of Recognizing Foreign, Hong Kong, Macao and Taiwan High-level Talents	2022	Guangdong Provincial People’s Government
	Notice on Further Implementation of the Preferential Policies on Individual Income Tax in Guangdong, Hong Kong and Macao Bay Area	2023	Guangdong Provincial Department of Finance
	Measures of Shenzhen Talent Residence	2023	Guangdong National People’s Congress Network
**Guangdong-Hong Kong-Macao Greater Bay Area Official Website**	Notice on the Declaration of High-end and Shortage Talents Enjoying Preferential Policies on Individual Income Tax in Hengqin Guangdong-Macao Deep Cooperation Zone	2023	Hengqin Guangdong-Macao Deep Co-operation Zone Economic Development Bureau
	Implementation Rules for Supporting Hong Kong and Macao Youth to Engage in Employment and Business Start-up in the Guangdong-Hong Kong-Macao Greater Bay Area	2022	Hong Kong and Macao Affairs Office
	Notice on the Implementation Rules (for Trial Implementation) for Encouraging and Supporting Innovation and Entrepreneurship of Hong Kong and Macao Youth in Guangzhou Nansha New Area (Free Trade Zone)	2020	Guangzhou Nansha Development Zone Hong Kong and Macao Cooperation Office

### 4.2 Stages of talent strategy development in the guangdong-hong kong-macao greater bay area

Taxation underpins national development as an essential lifeline. To operationalize the national directive “Notice on Preferential Policies on Individual Income Tax in the Guangdong-Hong Kong-Macao Greater Bay Area” (Tax [2019] No. 31),Successive policies were promulgated by the fiscal authorities of Guangdong Province and Guangzhou Municipality in 2019, 2020, and 2023, namely the “Preferential Individual Income Tax Policy Implementation Notice for the Guangdong-Hong Kong-Macao Greater Bay Area” (Guangdong Tax [2019] No. 2), the “Continuation Notice on the Implementation of Preferential Individual Income Tax Policies for the Guangdong-Hong Kong-Macao Greater Bay Area” (Guangdong Tax [2020] No. 29), and the “Further Implementation Notice on Preferential Individual Income Tax Policies for the Guangdong-Hong Kong-Macao Greater Bay Area” (Guangdong Tax [2023] No. 21). These notices are aimed at advancing the integration of tax policies within the region and facilitating the attraction and development of high-caliber talent.The policy has undergone initial formulation, subsequent redefinition of talent criteria, and subsequent refinement of implementation details, indicative of the policy’s ongoing intensification. Concurrently, a multidimensional analysis has been conducted, integrating the content of the Outline Plan and the promulgated landmark documents, stratifying the talent development in Guangdong-Hong Kong-Macao into the initial phase, the developmental phase, and the deep plowing phase.

The initial phase (2019). The Outline Plan was promulgated in 2019, with various governmental entities in Guangdong Province actively introducing an array of policies, including the “Regulations for Encouraging Industrial Development and Innovative Talents in Zhuhai” and the “Strategies for Fostering Hong Kong and Macao Talents’ Innovation and Entrepreneurship in Dongguan’s Songshan Lake”, with the objective of enticing talent to Guangdong and propelling the Greater Bay Area’s development.

The development phase (2020–2022). The benchmarks outlined in the Outline Plan indicate that by 2022, the Greater Bay Area is anticipated to markedly augment its overall capabilities and establish a framework for a globally premier bay area and a metropolis of international stature. Toward this objective, Guangdong-Hong Kong-Macao are accentuating talent development, incentivizing youth to pursue employment and entrepreneurial ventures in Guangdong, and enticing top-tier talents through the provision of tax incentives, housing stipends, and other policies.

The deep plowing phase (2023-Present). The long-term strategy delineated in the Outline Plan through 2035 envisions the Greater Bay Area constructing an economy centered on innovation and achieving substantial enhancements in economic and technological prowess, as well as global competitiveness. Concurrently, there will be an escalation in the quality of life for residents, societal civilization, and the soft power of culture to new heights. In 2023, policies including the “Guidelines for the Pilot Program on Establishing Criteria for Foreign ‘High-Precision, Top-Tier, and Scarce’ Talents in Guangzhou” and the “Protocol for the Identification of High-Level and Scarce Talents Eligible for Personal Income Tax Incentives in the Hengqin Guangdong-Macao In-Depth Cooperation Zone” were instituted. These directives underscore the development of sophisticated and elite talent to facilitate the high-quality advancement of the Greater Bay Area.

### 4.3 Determining the optimal number of themes

**Fig 2:**
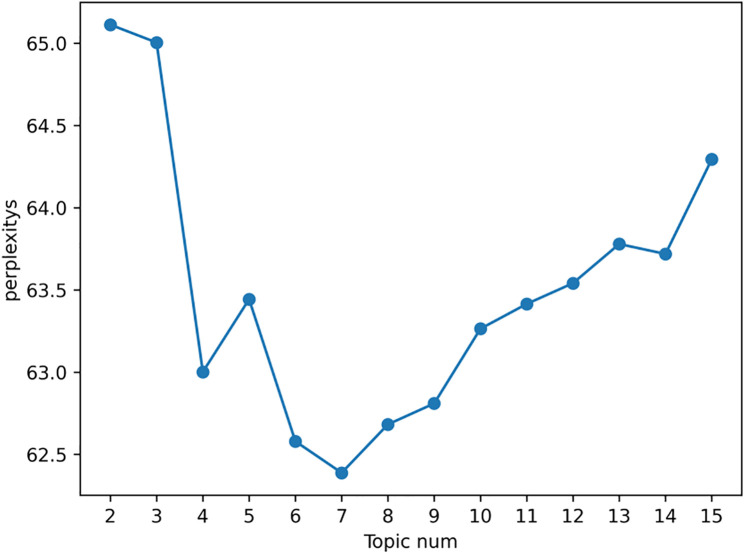
Calculation results of topic confusion.

**Fig 3:**
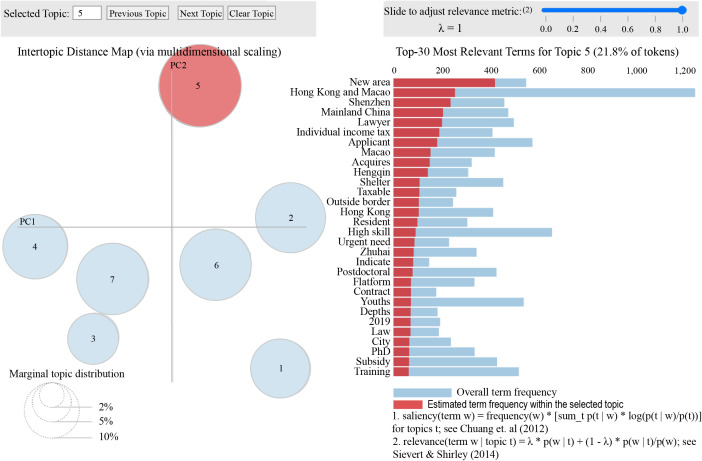
PyLDAvis result visualization.

The model was trained in a Python 3.9.16 environment running on Windows 11 (64-bit) with an Intel Core i7-12700H processor and 32GB RAM; for parameter tuning, we determined the optimal number of topics (k = 7) by calculating perplexity values for k ranging from 2 to 15 using `gensim.models.LdaModel`, with 1000 iterations performed for each k value—the perplexity curve (Fig 2) shows the minimum value at k = 7, and sensitivity analysis further confirmed the stability of the model (with hyperparameters set to α = 0.1 and β = 0.01) across 5 repeated training runs. We then used `pyLDAvis` (v3.4.1) to visualize topic boundaries (Fig 3), ensuring no overlapping themes by verifying that the cosine similarity between any two topics was less than 0.3. For result validation, we cross-validated the LDA-derived themes with TF-IDF keywords—extracted via `sklearn.feature_extraction.text.TfidfVectorizer`—and confirmed that the core terms of each theme (e.g., personal income tax for the “Hong Kong and Macao Tax Incentives” theme) matched the top TF-IDF keywords, thus ensuring thematic consistency.

## 5. Results

### 5.1 Thematic cross-sectional analysis

After the training of the LDA topic model is completed, we extract the probability distribution information of “topic-word” and “document-topic” generated by it. For the “topic-word” distribution, we organize the top 10 feature words with the highest probability in each topic, and judge and identify the content of each topic based on these feature words, and then name and categorize each topic. Finally, the results of theme summarization are divided into seven categories, as shown in Table 3.

**Table 3:** Distribution of “topic-word” in policy texts.

Number	Topic	Topic Keywords
1	Financial Talent Promotion	Financial; Hong Kong and Macao; Guangzhou; Advanced; Evaluate; Financial Institution; Internationalization; Doctor; Study Abroad;
2	Hong Kong and Macao Lawyers Settlement	Hong Kong and Macao; Solicitor;Shelter; Mainland China;Hong Kong; Taxation Services; Financial; Leading Figure; Settle Down; Guangzhou
3	High-skills Training	Training; High Skill; Bases; Applicant; Hong Kong and Macao; Young People; Fostering; Expert; Evaluations; Employment
4	Skill Development	High skill; Expert; Training; Cultivate; Security Bureau; Subsidize; Funding; Base; Professional Skills; Assessment
5	Hong Kong and Macao Tax Incentives	Dapeng New Area; Hong Kong & Macao; Shenzhen; Mainland; Lawyers; Individual Income Tax; Free Trade Zone; Subsidies; Hengqin; Concessions
6	Young talent Development	Hong Kong and Macao; Youth; Applicants; Promotion; Zhuhai; Guangdong, Hong Kong and Macao; Cultural Identity; Individual Income Tax; Highly Skilled; Base
7	Postdoctoral Training Grants	Funding; Postdoctoral; University; Education; Scientific and Technological Talent; Doctoral; Training; Leadership; Integration; Innovation

The establishment of a high-level talent hub has been articulated as a task requirement by General Secretary Xi Jinping for the development of the Guangdong-Hong Kong-Macao Greater Bay Area. The thematic distribution reveals that the Guangdong-Hong Kong-Macao talent strategy predominantly concentrates on scientific research, professional skills, legal expertise from Hong Kong and Macao, financial professionals, tax policies, and youth innovation and entrepreneurship, among other areas. Notably, there is an emphasis on integrating talents from Hong Kong and Macao into mainland regions, a strategy crucial for fostering deeper integration between Hong Kong, Macao, and the mainland. An analysis of thematic interrelations reveals that certain themes are intricately linked and exert mutual influence. For instance, the parallel advancement of scientific research and the cultivation of technical expertise, particularly among postdoctoral researchers, serves as a vital support and the central impetus for scientific and technological progress, which in turn is a key catalyst for the evolution of modern productive forces.

### 5.2 Theme heat analysis

**Fig 4:**
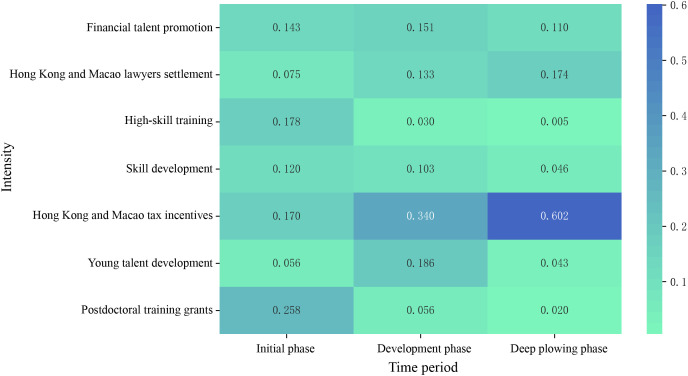
Heat map of thematic phases.

Drawing upon the findings from the LDA thematic modeling previously discussed, this section delineates distinct temporal intervals in accordance with the aforementioned overview of talent policies in Guangdong-Hong Kong-Macao. Each theme is allocated to its respective time window. Thereafter, the intensity of each theme within each time window is ascertained by normalizing the theme frequencies and graphically represents the fluctuations in thematic intensity utilizing heatmaps. The detailed analytical outcomes are portrayed in Fig 4. Observations derived from the heatmap reveal that:

#### 5.2.1 Initial phase (2019): A dual approach to research and skilled personnel.

Initially, the Guangdong-Hong Kong-Macao Talent Strategy centers on the recruitment of scientific and technical talents. Within this framework, research talents pertain to individuals with doctoral and post-doctoral qualifications, whereas skill talents relate to those engaged in high-skill training and skill development. Guangdong, a vanguard of China’s manufacturing prowess, aims to bolster its pool of highly educated talents and enhance skills, thereby spearheading the high-quality development of the Guangdong-Hong Kong-Macao Greater Bay Area through team-building initiatives. In conjunction with the thematic heat analysis, postdoctoral training grants (0.258), high-skill training (0.178), and Hong Kong and Macao tax incentives (0.170) emerge as central components of the strategy. At this juncture, the talent strategy embraces a dual strategy of “cultivation and concurrent introduction,” pursuing a bifurcated approach that addresses both scientific research and skill development, and constructs a policy framework characterized by “governmental macro-control and enterprise-level autonomy in employment” to draw talents from across the nation to Guangdong, exhibiting structured, standardized, and scientific attributes.

The scale of doctoral and post-doctoral research talent in Guangdong Province has surpassed 50,000, positioning it at the forefront nationally. Conversely, in 2015, the province’s doctoral enrollments totaled a mere 3,225, ranking it fourth nationally. Upon comparing the doctoral enrollments of Beijing, Shanghai, Guangdong, and Jiangsu, it is observed that Beijing significantly outpaces with 21,302 enrollments, equating to 6.6 times Guangdong’s enrollment in that year, nearly 40% of Guangdong’s current doctoral and post-doctoral talent pool, and constituting 29% of the national PhD student population. In contrast, Guangdong’s efficacy in nurturing scientific research talent lags slightly, particularly when juxtaposed with Shanghai’s 6,336 and Jiangsu’s 6,570 enrollments, which are nearly double that of Guangdong’s—a discrepancy ill-suited to Guangdong’s stature as an economic juggernaut nationally [43]. During the 2018 National People’s Congress, Luo Jun, President of Sun Yat-sen University, advocated for an expansion in the number of doctoral enrollments at Guangdong’s institutions of higher education, highlighting the significance of scale and quality of PhD holders for national research and innovation. Data indicate that Guangdong’s doctoral acceptance rate (0.33 per 10,000 individuals) and the ratio of PhD students to researchers (4%) fall below the national average (0.54 per 10,000 individuals, 6.5%), with a pronounced disparity in comparison to locales such as Beijing, Shanghai, and Suzhou [44]. Against this backdrop, the 2019 Outline Plan has proposed the establishment of a talent highland for Guangdong-Hong Kong-Macao. Subsequently, the Guangzhou Municipal Government promulgated the “Opinions on the Implementation of the ‘Guangju Talent Plan’” and “Opinions on Accelerating the Innovation and Development of Doctoral and Postdoctoral Talents in the New Era,” with objectives to expedite the recruitment and development of high-caliber talents and to bolster the research and development and innovation capabilities of Guangdong’s enterprises.

Skilled personnel in Guangdong, despite the province’s status as China’s manufacturing hub, are insufficient in both number and quality to satisfy its developmental demands. Primarily, the issue is rooted in the scale of vocational training. The proportion of graduates from intermediate and advanced vocational institutions in the Pearl River Delta region remains below 10%, and the figure for relevant graduates from Hong Kong and Macao is sparser still. Secondly, when examining the quality dimension, the talent structure within higher vocational education in the Guangdong-Hong Kong-Macao Greater Bay Area exhibits imbalances, and a dearth of high-quality educational resources for vocational training hinders effective response to the pressing demands of China’s manufacturing industry’s transformation and upgrading [45]. General Secretary Xi Jinping has emphasized that the skilled labor force is pivotal in propelling the shift from “Made in China” to “Created in China,” and is instrumental in fostering the economy’s high-quality development [46]. Concurrently, aligning with the “capital-skill” complementary theory, it is imperative for high-skilled personnel to bolster the innovation capacity of enterprises. The government augments the supply of high-skilled labor via talent introduction policies, refines the labor structure within enterprises, and achieves a synergistic effect [47]. In light of this, the Party Central Committee places significant emphasis on enacting a suite of policies, including the “Guangdong Province Vocational Skill Enhancement Action Implementation Plan (2019-2021)” and “Guangdong Province Vocational Skill Enhancement Training Subsidy Claims Management Measures,” employing subsidies, incentives, and safeguards to attract high-skilled talent, thereby upgrading skill levels and expanding the contingent of high-skilled personnel.

#### 5.2.2 Development phase (2020–2022): Base construction to promote the integration of youth from Guangdong, Hong Kong and Macao.

To facilitate the assimilation of Hong Kong and Macao into the national development blueprint, the Guangdong Provincial Government has promulgated the Implementation Plan for Establishing Innovation and Entrepreneurship Hubs for Hong Kong and Macao Youth, with the objective of establishing bases across nine cities in the Pearl River Delta by 2025, and instituting a “1 + 12+N” incubation framework [48]. Nonetheless, against the backdrop of “one country, two systems, and three jurisdictions”, the youth of Hong Kong and Macao exhibit a limited comprehension of mainland policies. The prevailing talent strategy emphasizes the facilitation of industrial integration, the integration of resources across the three regions, and the construction of a multi-functional demonstration platform. Hong Kong and Macao Tax incentives, along with the Young talent development, possess a heat index of 0.340 and 0.186, respectively, reflecting the concentrated, integrated, and youthful nature of the talent strategy.

The young talents development and the base construction are interdependent and mutually reinforcing. In 1920, Marshall introduced the agglomeration effect theory, highlighting that talent concentration can diminish the costs associated with labor and knowledge dissemination, and can foster the development of high-quality urban infrastructure and enhance the innovation efficiency of enterprises [49]. Within the framework of the Guangdong-Hong Kong-Macao Greater Bay Area’s development, the youth from Hong Kong and Macao play pivotal roles. The survey indicates that 78.5% of Hong Kong and Macao’s youth possess an inadequate understanding and vague awareness of Guangzhou’s entrepreneurial ecosystem, including incubators and other platforms. A mere 16.8% consider them to be significantly beneficial, while 15% deem them unhelpful. Furthermore, approximately 90% of Hong Kong and Macao’s youth in Guangzhou report not having access to comprehensive social security benefits, and a small fraction are eligible for limited social security benefits, including medical and workers’ compensation insurance, attributed to their parents’ occupational, educational, or residential status on the mainland [50]. On a global scale, Shenzhen was ranked eighth in the employment preferences of Hong Kong’s professionals in 2018, whereas Singapore maintained its leading position. Australia, New Zealand, and several European and North American countries and regions are also preferred by Hong Kong’s professionals and have emerged as favored destinations for overseas employment and settlement [51]. This exodus of Hong Kong’s professionals has precipitated a significant brain drain within the Guangdong-Hong Kong-Macao Greater Bay Area, presenting formidable challenges to the establishment of an international science, technology, and innovation hub in the region. In response to this scenario, General Secretary Xi Jinping emphasized leveraging the Guangdong-Hong Kong-Macao cooperation platform to entice young individuals from Hong Kong and Macao to pursue education, employment, and residence in the mainland, thereby fostering a stronger sense of national identity. In 2020, a concerted effort among Guangdong-Hong Kong-Macao led to the promulgation of several policies, including the “Guangdong Province Regulations on Fostering Employment and Entrepreneurship for Youth from Hong Kong and Macao in the Nine Cities of the Guangdong-Hong Kong-Macao Greater Bay Area,” “Guidelines for the Declaration of Subsidies for Innovation and Entrepreneurship for Youth from Hong Kong and Macao Arriving in Guangzhou,” “Macao Youth Policy (2021-2030),” along with Hong Kong’s Policy Address, among others. These policies are designed to incentivize youth from Hong Kong and Macao to engage in employment and initiate businesses within the Greater Bay Area’s cities through employment subsidies, startup grants, and other measures. For instance, the Hong Kong “Greater Bay Area Youth Employment Initiative” offers 2,000 positions, with the government providing a monthly stipend of HK$10,000 to graduates for up to 18 months [52]. These initiatives are designed to entice youth from Hong Kong and Macao to foster cross-boundary development within the Greater Bay Area and to ignite their entrepreneurial aspirations. The year 2024 will mark the preliminary establishment of the “1 + 12+N” network of innovation and entrepreneurship hubs for youth from Hong Kong and Macao in the Greater Bay Area, facilitating the cultivation of nearly 4,000 projects from Hong Kong and Macao, and attracting 5,500 youth from Hong Kong and Macao into employment. This underscores the significant emphasis the State places on nurturing young talents and establishing foundational infrastructure.

#### 5.2.3 Deep plowing phase (2023-present): Integration of systems across domains to facilitate mobility of talent.

The release of the Outline Plan marks its fifth anniversary. The Guangdong-Hong Kong-Macao Greater Bay Area, a distinctive economic nexus characterized by “one country, two systems, and three tariff zones,” is unparalleled internationally. By integrating the heat and temporal dynamics of the themes, the Hong Kong and Macao tax incentives and the Hong Kong and Macao lawyers settlement possess heat values of 0.602 and 0.174, respectively, exhibiting a consistently ascending trend. This reflects the vertical and in-depth developmental trajectory of the state’s construction in this region. As the theme represents a salient issue across various developmental stages, this paper aims to more effectively discern the thematic evolution. This study employs the LDA model to derive the “theme-word matrix” for each phase, identifying keywords with the highest likelihood of occurrence during the three strategic phases, and endeavors to analyze the thematic evolution across different stages with fine-grained precision, revealing that the current talent strategy is marked by synergistic, international, and digital attributes.

**Fig 5:**
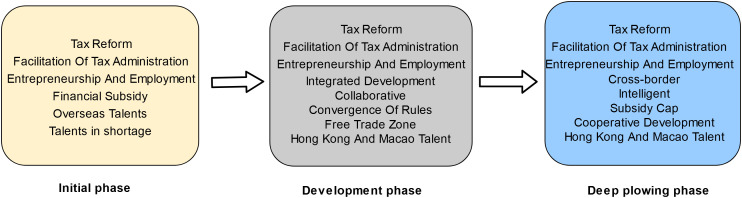
Evolutionary trend of keywords for hong kong-macao tax benefits topic.

Tax incentives have consistently been a central concern in the developmental trajectory of the Guangdong-Hong Kong-Macao Greater Bay Area. Fig 5 illustrates that the stable core keywords for Hong Kong and Macao tax incentives across various developmental stages include “tax reform,” “entrepreneurship and employment,” and “facilitation of tax administration,” reflecting widespread interests. Upon examination of the specific terms at each stage, it is observed that the thematic focus of Hong Kong and Macao tax incentives has evolved through a developmental sequence from individual tax subsidies to the consolidation of three major real estate taxes, and finally to tax digitization, which aids in fostering regional collaboration between Guangdong-Hong Kong-Macao. In 2019, the State Administration of Taxation (SAT) promulgated a circular regarding preferential policies on individual income tax for the Guangdong-Hong Kong-Macao Greater Bay Area (Tax [2019] No. 31), designed to expedite the influx of talent and offering special tax deductions for overseas high-level and scarce talents in the Greater Bay Area who have received preferential policies. This is achieved by offering local financial subsidies for the surplus personal tax (the portion of taxable income exceeding 15%) in the nine cities of the Pearl River Delta. Upon entering the developmental phase, the tax system, regulations, and institutional barriers within the Greater Bay Area impede further integration [53], rendering the alignment of tax regulations a critical task. The Guangdong Free Trade Area acts as a cooperative conduit, enhancing collaboration between the Mainland, Hong Kong, and Macao. From 2021 to 2022, the programs for Hengqin (Guangdong and Macao), Qianhai (Shenzhen and Hong Kong), and the Guangzhou Nansha Cooperation Zone were successively released by the Central Committee and the State Council. These programs explicitly emphasize the need to strengthen the alignment of the Free Trade Zone with the rules of Hong Kong and Macao, thereby offering new avenues for the integration of regulations within the Greater Bay Area. The tax policies of the three free trade zones, which focus on employment and entrepreneurship tax arrangements for Hong Kong and Macao talents, vary based on geographic location. Specifically, Nansha benefits Hong Kong and Macao residents, Qianhai targets Hong Kong residents, and Hengqin covers both domestic and foreign high-end talents as well as Macao residents. In 2023, the “Several Measures on Taxation to Support the High-Quality Development of Guangdong” were launched by the Guangdong Provincial Taxation Bureau. These measures focus on the construction of the Greater Bay Area and aim to promote the unification and standardization of rules on tax collection, administration, and services through mutual recognition of tax-related credits, the establishment of smart terminals, and the integration of services. Additionally, they introduce the innovative “cross-border RMB full-electronic tax payment” service, addressing the tax payment challenges faced by non-resident taxpayers. The innovative “cross-border RMB full-electronic tax payment” service has been widely praised for solving these challenges. Enhanced cross-border cooperation through the introduction of “Guangdong Smart Assist” self-service kiosks in Macao has enabled cross-border self-service across more than 200 livelihood services. The tax authorities in Guangdong and Shenzhen have initiated the “Bay Area Through Service” initiative, integrating the e-Tax Bureau, self-service, and remote tax handling systems to facilitate the cross-regional handling of over 300 tax and fee-related businesses. These initiatives have reaffirmed the requirement outlined in the Opinions on Further Deepening Tax Administration Reform, issued by the State Council, which stipulates that by 2023, a new system of tax and fee services, characterized by “no dead ends offline, no closure online, and extensive customized coverage,” will be substantially completed, and services will evolve from standardization towards refinement, intelligence, and personalization.

**Fig 6:**
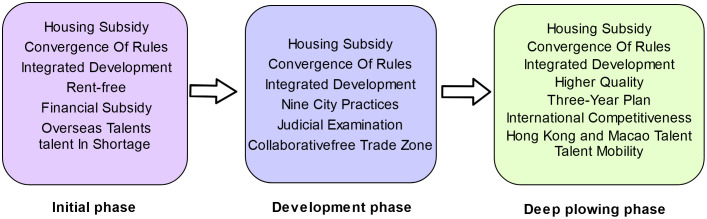
Evolutionary Trend of keywords for lawyers’ settlement themes in Hong Kong and Macao.

Within the framework of “one country, two systems,” the collaborative development of the legal service industry among Guangdong-Hong Kong-Macao encounters challenges, including differences in legal systems and a lack of mutual recognition of practicing qualifications. The 2019 Outline Plan underscores the importance of enhancing cooperation between Guangdong-Hong Kong-Macao in advancing the legal service industry. The Guangdong Provincial Party Committee and Government issued the “Implementation Opinions on the Outline Plan for the Guangdong-Hong Kong-Macao Greater Bay Area’s Development,” which emphasizes the convergence of rules, eliminates institutional barriers, and facilitates the movement of factors. In 2020, General Secretary Xi Jinping highlighted the necessity of capitalizing on the construction of the Guangdong-Hong Kong-Macao Greater Bay Area to advance the convergence of rules and the docking of mechanisms, as well as to elevate the level of market integration [54]. Given this context, the “convergence of legal rules in the Guangdong-Hong Kong-Macao Greater Bay Area” has emerged as a focal point of discussion. As illustrated in Fig 6, the housing policy for Hong Kong and Macao lawyers centers around the core contents of “housing subsidy,” “convergence of rules,” and “integrated development” at various stages. To attract Hong Kong and Macao lawyers to the Mainland for development, the State facilitates the synergy of legal policies in the Bay Area by offering housing, education, and other services to promote high-quality growth [55]. Given the disparities in legal systems and legal terminologies among the Mainland, Hong Kong, and Macao, it is imperative to dismantle legal system barriers, promote the collaborative development of lawyers, establish a business environment grounded in the rule of law, and foster the high-quality development of Guangdong-Hong Kong-Macao. In 2020, the State Council’s General Office issued the Pilot Measures for Lawyers Practicing in the Guangdong-Hong Kong-Macao Greater Bay Area, enabling Hong Kong and Macao lawyers to practice in nine mainland cities. In July 2021, a successful first licensing examination was conducted, attracting over 700 applicants and achieving a pass rate of over 70% [56]. This surpassed the average pass rate of the National Legal Profession Qualification Examination for Hong Kong and Macao Residents, indicating significant progress in opening up the Mainland’s legal services market to Hong Kong and Macao lawyers. Subsequently, the Guangdong Provincial Department of Justice facilitated the implementation of various measures, including the establishment of incubation centers for Hong Kong and Macao lawyers in Nansha, Guangzhou, the development of a plan for the high-quality growth of the legal profession in Shenzhen, and the establishment of a public legal service center in Zhuhai focused on foreign affairs. These measures aimed to attract legal services from Hong Kong and Macao lawyers within the greater bay area. These initiatives have promoted the alignment of legal service industry regulations among Guangdong-Hong Kong-Macao, and have collectively enhanced the capacity and international competitiveness of legal services in the region.

### 5.3 Thematic content evolution analysis

**Fig 7:**
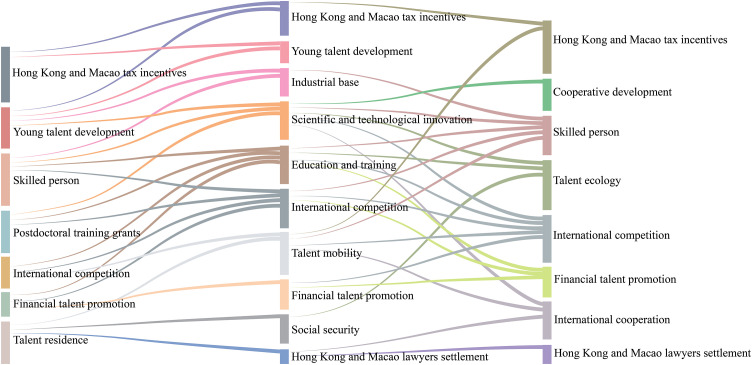
Evolutionary path of Guangdong, Hong Kong and Macao talent strategy themes.

Refining the path analysis of topic evolution necessitates a preprocessing step, involving the exclusion of topics with low heat, prior to assessing topic similarity. Experimental data demonstrate that optimal results are achieved when the heat threshold is established at 0.025. Further refinement of the themes was accomplished using a similarity threshold of 0.3 [57]. Ultimately, a Sankey diagram illustrating the evolution trajectory of the themes related to the talent strategy of Guangdong-Hong Kong-Macao was produced, and the outcomes are presented in Fig 7.

#### 5.3.1 Focus on the construction of a multifaceted talent ecosystem.

With regard to thematic relevance, there is a close association between the talent ecology and education and training, social security, as well as science and technology innovation, exhibiting substantial developmental significance. Upon analyzing the evolutionary trajectory, it is evident that the emergence of the talent ecology during the deep cultivation period indicates a positive trend towards deep integration of these three elements within the Greater Bay Area in the future. This trend underscores the necessity for the Greater Bay Area to move towards comprehensive integration, aiming to foster a harmonious regional talent ecosystem. Social security, as a foundational system, safeguards people’s livelihoods, regulates income distribution, and fosters social equity [58]. Conversely, universities offer talent support for scientific and technological advancements, which in turn facilitate industrial upgrading and transformation, leading to the creation of new economic growth points and enhancing the competitiveness of the Greater Bay Area. This further validates the talent policy outlined in the 20th CPC National Congress report, wherein the integration of education, science and technology, and talent is emphasized, indicating a fresh direction for talent development in the new era [59]. Drawing upon the results of theme similarity calculations, the diversified interplay within the talent ecology primarily centers around the following domains:

Talent ecology and education. Education serves as a pivotal aspect in evaluating the talent ecology. Serving as the cornerstone for cultivating innovative talents and transforming scientific and technological advancements, colleges and universities facilitate industrial upgrading and economic development through “industry-university-research” collaboration, infusing new momentum into the Bay Area. The three prominent Bay Areas globally are distinguished by their abundant higher education resources, including the New York Bay Area featuring Columbia University and other renowned universities, the San Francisco Bay Area housing Stanford University, and the Tokyo Bay Area boasting over 120 institutions of higher learning, among them the University of Tokyo. Conversely, the premium university resources in the Guangdong-Hong Kong-Macao Greater Bay Area are predominantly concentrated in Hong Kong, which, while internationally competitive, lags behind the world’s leading Bay Areas in terms of higher education ecology [60]. Specifically, despite its large population, the Greater Bay Area exhibits a low proportion of highly educated individuals and a scarcity of universities and world-renowned schools. Moreover, during its economic transformation, the Greater Bay Area possesses a limited proportion of highly educated talents, resulting in a significant disparity between the supply and demand for talents in science and technology innovation and industrial upgrading. In contrast to the world’s three Bay Areas, which boast the highest proportion of talents with bachelor’s degrees, the Guangdong-Hong Kong-Macao Bay Area exhibits a significantly lower proportion of talents with lower education levels, particularly PhDs. Despite being a core innovation city with a substantial number of talents holding bachelor’s degrees, Shenzhen’s proportion of PhDs remains below 2%, falling short of the Greater Bay Area’s average and warranting urgent improvement [61].

Talent ecology and social security. Social security encompasses various aspects of daily life, including settlement, residence, education, medical care, and pension, and is pivotal to the creation of a habitable environment in the Greater Bay Area. A comprehensive social security service can elevate the quality of life of residents, foster a heightened sense of well-being, and entice top-tier talent to the Greater Bay Area. This aligns with Glaeser’s theory of the “Consumer City,” which emphasizes that high-quality urban services and a comfortable environment are essential for attracting skilled labor [62]. However, when compared to the world’s three largest Bay Areas, the Guangdong-Hong Kong-Macao Greater Bay Area still has areas for enhancement in terms of daily living conditions. Constrained by multiple factors, some cities in the region exhibit notable deficiencies in public services such as healthcare, pension, and housing, which hinders the region’s ability to attract skilled labor [60]. In Shenzhen, for example, real estate prices are elevated, homeownership rates remain low, the availability of educational resources is tight in some cities, and issues related to aging are severe. To attract global talent, the Greater Bay Area must address these deficiencies in daily living conditions, tackle the challenges of employment, population density, transportation, and housing prices, and provide a comprehensive development platform to enhance the retention of skilled labor.

The talent ecology is closely linked with scientific and technological innovation. Talent drives progress in science and technology, which in turn spurs transformation and upgrading across the Greater Bay Area, cementing its status as a hub for top-tier talent. According to the 2022 Guangdong-Hong Kong-Macao Greater Bay Area Science and Technology Achievement Report, the region has attracted nearly 9,000 overseas talents specialized in innovation, indicating that the number of full-time academicians in Guangzhou has risen to 64 by 2021. The incorporation of academics from Hong Kong and Macao has substantially bolstered the region’s capabilities in both fundamental and original innovation. The Greater Bay Area is witnessing a net inflow of skilled labor, high-skilled digital professionals, and other talent [63]. The San Francisco Bay Area is recognized globally as a model for high-tech research and development and fostering innovative talent, hosting some of the world’s preeminent academic institutions and research facilities. The region not only attracts millions of scientific and technological elite, notably including over 30 Nobel Laureates, but also establishes a luminous edifice of knowledge and wisdom. Silicon Valley, serving as its core, boasts a high level of education, with 22% of the workforce possessing a master’s degree or higher, 49% holding a bachelor’s degree or higher, and over 40% of the technical staff originating from overseas, exemplifying diversified and internationalized traits. In contrast, there is a need for the Guangdong-Hong Kong-Macao Greater Bay Area to enhance its population’s education level and global talent influence. The percentage of entrepreneurial talent serves as one of the most significant indicators of regional vitality among the world’s three largest Bay Areas. However, the Guangdong-Hong Kong-Macao Greater Bay Area does not rank among the top in this regard, with the percentage of entrepreneurial talent falling below 8%, which is substantially lower than the San Francisco Bay Area’s level of over 13%. Despite being a leading city in the Greater Bay Area with strong talent attraction, Shenzhen’s proportion of entrepreneurial talent has remained below 8% [64]. Recently, the rise of the information technology industry has been observed in Guangdong-Hong Kong-Macao, positioning itself in the new technological revolution. High-tech industries have emerged as the backbone of the economy, continuing to spearhead advancements in the future. This reinforces the industry’s foundation and elevates its position on the global technological map.

#### 5.3.2 Talent Mobility in the Bay Area Embedded in International Competition and Cooperation.

In terms of the evolutionary path of the theme, talent mobility becomes a prominent focus during the development stage, while the theme of international competition pervades throughout the development of the Guangdong-Hong Kong-Macao Greater Bay Area, reflecting the expansion of the scope of talent mobility in the Bay Area from China to the world. This aligns with the goal of the Outline Plan to build a first-class Bay Area with international competitiveness. This paper analyzes the evolutionary logic of the themes of talent mobility, international cooperation, and competition, with the aim of revealing their significance for the development of the Bay Area.

Bay Area Talent Mobility and International Competition. The Guangdong-Hong Kong-Macao Greater Bay Area, as a convergence of innovative and internationalized talent in the Asia-Pacific region, recorded a cross-provincial net inflow of 826,100 people in 2019, substantially bolstering its labor resources. However, the proportion of foreign professionals who have made Guangdong their long-term residence remains low [65]. Consequently, the Ministry of Finance (MOF) has launched a personal tax subsidy policy to effectively reduce the disparity in tax burden between the Mainland and Hong Kong, essentially exempting eligible expatriate individuals from personal income tax. The policy aims to reduce the tax burden of expatriate individuals, increase their appeal to the Mainland working environment, and promote talent mobility. Coupled with the Central Government’s clear mandate for Shenzhen to implement enhanced and streamlined procedures for the admission and exit-entry administration of highly skilled foreign professionals, this series of initiatives signals that Guangdong Province is becoming increasingly attractive to highly skilled foreign professionals. In the same year, there was a net inflow of 30,000 people into Hong Kong, reflecting the strong competition for talents [66]. Economists have long recognized that talent mobility is a core driver of regional economic development. Leading economists in the field of new growth theory, such as Paul Romer and Robert Lucas, have emphasized that the concentration of talent and its drive for innovation are crucial for economic growth [67]. The Guangdong-Hong Kong-Macao Greater Bay Area has directly promoted the accumulation of talent through the implementation of the talent admission policy. Despite some progress being made in terms of talent mobility, the Bay Area still faces challenges compared with global benchmarks, such as the international talent mobility mechanism remaining incomplete and the talent mobility environment needing improvement, which to a certain extent hamper the advancement of the Bay Area’s international standing.

Bay Area Talent Mobility and International Cooperation. Talent mobility not only promotes the pooling and acceleration of human capital but also exerts a significant impact on international exchanges and cooperation. By leveraging scientific and technological innovation resources and advantageous industries, the Guangdong-Hong Kong-Macao Greater Bay Area facilitates the introduction of technology, capital, and market opportunities, thereby deepening talent interaction and cultural integration and broadening the path of its internationalization. Specifically, the Bay Area ought to enhance technological cooperation and cross-border capital flows. Furthermore, an open investment policy should be implemented, establishing an efficient capital deployment mechanism to attract international capital and provide financial support and a market stage for innovation and entrepreneurship. For instance, the Greater Bay Area Science Forum 2023 focused on innovative development strategies and technological collaboration, covering cutting-edge areas such as artificial intelligence, green energy, and blockchain. It emphasized the importance of international cooperation and called for addressing global challenges, accelerating the building of a carbon-neutral and ecological civilization. Therefore, scientists suggested the establishment of a special fund for research funding to deepen cross-sector collaboration, providing an excellent opportunity for international cooperation and development in the Guangdong-Hong Kong-Macao Greater Bay Area.

### 5.4 Talent policy effectiveness evaluation

Amidst the acceleration of globalization and regional integration, the talent strategy of the Guangdong-Hong Kong-Macao Greater Bay Area, a vital driver of China’s economic growth, is crucial for sustainable development. Talents are central to economic transformation and innovation. Their mobility, retention, and policy feedback significantly influence the global competitiveness of the Greater Bay Area. This study constructs a policy evaluation system that focuses on indicators of talent inflow, satisfaction, and economic and social impacts. It quantitatively assesses the effects of talent policies, explores the interaction mechanism between policies and talent behaviors, and provides empirical evidence along with prospective suggestions for optimizing Greater Bay Area policies. These efforts aim to establish the region as an international talent hub and promote economic prosperity and social progress.

#### 5.4.1 Number of talent inflows.

Talent policies can be categorized into four main types: the first type focuses on “attracting” and “retaining” talent through introduction and protection; the second type emphasizes “nurturing” talent through cultivation and development; the third type centers on talent management and evaluation mechanisms; and the fourth type highlights regional differences where talent introduction surpasses talent cultivation, as observed in Guangdong and Sichuan. These policies frequently integrate material and spiritual incentives with attractive infrastructure development. This approach directly or indirectly promotes regional talent concentration and creates favorable conditions for cross-border, cross-regional, cross-departmental, and cross-level talent mobility. By comparing talent inflow data before and after policy implementation, these efforts offer crucial references for assessing policy effectiveness.

**Fig 8:**
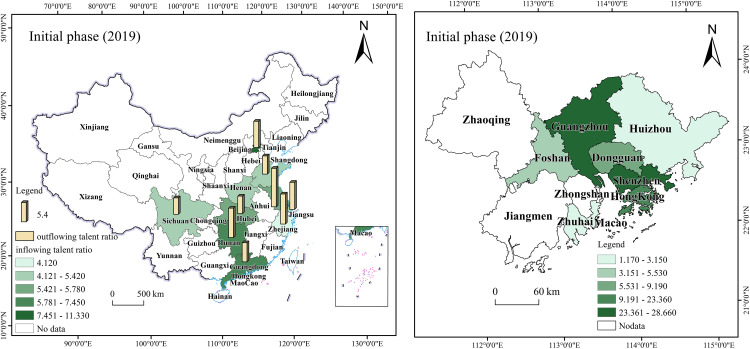
Initial phase of the Guangdong-Hong Kong-Macao Greater Bay Area talent flow map.

During the initial phase, talent flow in the Guangdong-Hong Kong-Macao Greater Bay Area was primarily concentrated in Guangzhou and Shenzhen, demonstrating clear geographical characteristics, as shown in Fig 8 [68]. In 2019, talent inflow into the Greater Bay Area mainly came from Beijing (11.33%), Hunan (7.45%), and non-Pearl River Delta regions within Guangdong Province (6.99%). Meanwhile, talent outflow from the Greater Bay Area mainly went to Jiangsu (10.71%), Zhejiang (8.35%), and Hunan (8.18%). This shows that the Greater Bay Area had the most frequent two-way talent interactions with other Guangdong cities and neighboring Hunan Province. During this period, cities in the Greater Bay Area introduced a series of talent-attracting policies. For example, Shenzhen’s Peacock Program and Guangzhou’s Guangzhu Talent Program provide financial support and housing subsidies for imported talents. Regarding industrial development strategies, policymakers prioritized promoting local advantageous industries, such as electronic information and finance, and established industrial parks to attract professionals in these fields.

In terms of talent distribution, the Guangdong-Hong Kong-Macao Greater Bay Area exhibits high concentration in Shenzhen (28.66%), Guangzhou (26.69%), and Hong Kong (23.36%). The overall net inflow ratio stands at 1.39%, reflecting a notable talent concentration effect. Notably, 70% of the talent inflow was concentrated in Guangzhou and Shenzhen, establishing them as the core areas for talent flow within the region. While the policy has effectively attracted external talent, internal talent mobility within the Greater Bay Area remains relatively limited. In addition to Guangzhou and Shenzhen, other Pearl River Delta cities demonstrate weaker capabilities in attracting and retaining talent. This indicates that the Greater Bay Area needs to further optimize its internal talent structure to facilitate the rational mobility and balanced development of talents within the region.

**Fig 9:**
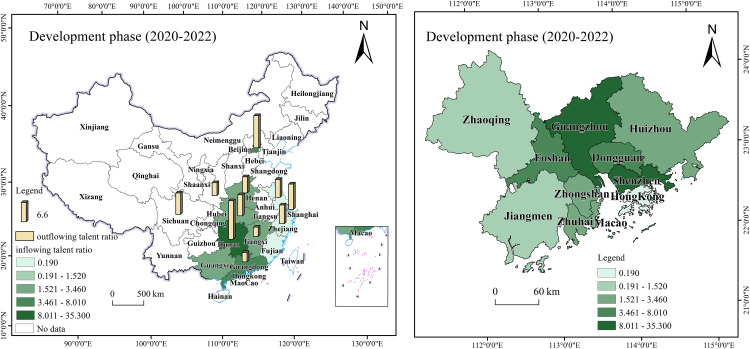
Development phase of the Guangdong-Hong Kong-Macao Greater Bay Area talent flow map.

During the development phase, talent flow in the Guangdong-Hong Kong-Macao Greater Bay Area presents new characteristics, as shown in Fig 9 [69]. From 2020 to 2022, the primary sources of talent inflow were Hunan (12.91%), Beijing (8.28%), Jiangxi (6.28%), and Hubei (5.96%). The study indicates that the Greater Bay Area has successfully attracted a large number of talents, largely due to its diverse industrial structure and well-developed infrastructure. Particularly following the release of the Plan Outline, the talent flow in the Greater Bay Area shifted from “supply exceeding demand” to “demand exceeding supply”. As more talents influx, job market competition intensifies, making brain drain to some extent inevitable. Specifically, apart from their home provinces, the talent outflow and inflow of the Greater Bay Area remained relatively balanced. Hunan Province was the most frequent talent flow destination, accounting for 13.2%, followed by Beijing (11%), Shanghai (8.7%), and Sichuan (7.3%). While Hunan Province remains the most active in two-way talent flow within the Greater Bay Area, the proportion of talent heading to Beijing and Shanghai has increased compared to the inflow. This indicates that while the Greater Bay Area has achieved significant success in attracting talent, it also faces the challenge of brain drain. This implies that the precision and effectiveness of relevant policies need to be further enhanced.

Meanwhile, as the 9 + 2 city cluster of the Guangdong-Hong Kong-Macao Greater Bay Area continues to develop, communication and exchanges between the Greater Bay Area and major cities are deepening. This will undoubtedly further enhance the Greater Bay Area’s talent attractiveness. To ensure a balanced talent structure across the Greater Bay Area’s regions, talent policies in this stage continue to be refined, providing mobile talents with a range of safeguards, including children’s education, housing, healthcare, and social insurance, to better meet their needs. Additionally, as transportation infrastructure improves and industrial synergy among cities strengthens, the formation of complete industrial chains and clusters in each region effectively promotes smoother and more rational talent flow within the Greater Bay Area. According to the report, in terms of talent distribution, Shenzhen’s share increased to 35.30%, Guangzhou’s rose to 31.32%, and neighboring cities like Dongguan (8.01%), Foshan (7.1%), and Huizhou (3.46%) also experienced increases.

**Fig 10:**
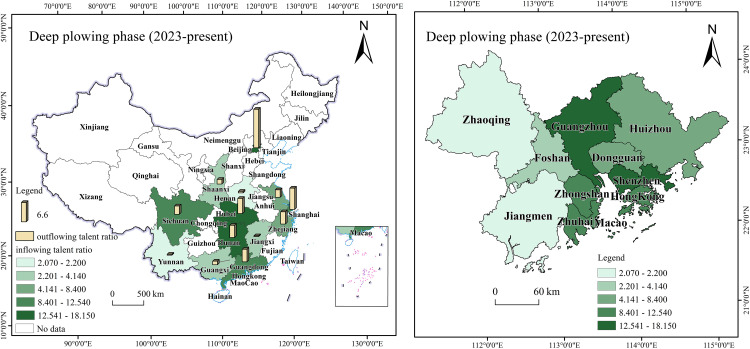
Deep plowing phase of the Guangdong-Hong Kong-Macao Greater Bay Area talent flow map.

During the deepening phase, as the construction of the Guangdong-Hong Kong-Macao Greater Bay Area progresses, regional governments are committed to optimizing the talent policy system. They improve the quality of life for talents through comprehensive housing, education, and healthcare support, enhancing the Greater Bay Area’s appeal and diversifying talent inflow sources. As shown in Fig 10 [70], from 2023 onward, the Greater Bay Area has displayed diversified trends in talent inflow sources and industries. The intended inflow of talent from first-tier and new first-tier cities accounts for 19% and 46%, respectively. Talent inflow primarily originates from Beijing (12.8%), Hunan (10%), Hubei (8.9%), Shanghai (6.2%), Chongqing (6.1%), and Yunnan (2.2%).

Concurrently, the Greater Bay Area’s industrial transformation, upgrading, and science and technology innovation policies have guided talent toward emerging industries and high-end manufacturing, prompting adjustments in the region’s talent distribution. Zhuhai (11.92%), Huizhou (7.56%), and Zhongshan (10.47%) have attracted greater talent inflows due to industrial development opportunities. To deepen Guangdong-Hong Kong-Macao integration, the talent proportion from Hong Kong and Macao rebounded to 12.54% and 8.4%, respectively. Shenzhen (18.15%) and Guangzhou (17.62%) continued to play pivotal roles. Regional collaborative development policies promote the free flow of factors among Greater Bay Area cities and talent movement influenced by industrial layout and personal development needs, fostering diversified and balanced talent flows.

#### 5.4.2 Talent satisfaction.

Based on the LDA thematic model results, this study designs a questionnaire encompassing four dimensions: tax incentives, housing security, employment support, and entrepreneurship support. A satisfaction survey targeting high-end, young, and skilled talents in the Guangdong-Hong Kong-Macao Greater Bay Area was conducted via stratified sampling and interviews. The findings are presented in Table 4.

**Table 4:** Talent satisfaction.

Evaluation dimension	very satisfied	satisfied	general	dissatisfied	Very dissatisfied
Tax Incentives	40%	35%	15%	9%	1%
Housing Security	30%	25%	20%	20%	5%
Employment Support	33%	42%	15%	5%	5%
Entrepreneurship Support	35%	35%	15%	10%	5%

First, tax incentives policy. The individual income tax incentives in the Greater Bay Area cap the effective tax burden of high-end and regionally scarce talents at 15% through fiscal subsidies. This program has substantially optimized the tax burden environment for talents in the Greater Bay Area and plays a crucial role in inter-regional talent competition. Survey results indicate that 75% of respondents were satisfied with the policy, acknowledging its positive impact on reducing the tax burden and increasing income levels, which has effectively attracted high-income talents. However, the 25% dissatisfaction or very dissatisfaction feedback indicates there is room for enhancing policy promotion, simplifying application procedures, and broadening coverage. Some talents may miss policy benefits due to information asymmetry or be deterred by complex application procedures. Furthermore, some high-income groups consider the subsidies inadequate to fully offset their tax burden.

Second, housing security policy. Several cities in the Guangdong-Hong Kong-Macao Greater Bay Area have implemented housing protection policies for talents, such as Shenzhen’s secure tenure policy. This policy provides eligible talents with housing subsidies and talent apartments to alleviate housing pressure. However, the survey reveals a satisfaction rate of only 55% in this dimension, indicating policy deficiencies in supply-demand matching and precision. The primary issues are tight housing supply and stringent application requirements. Existing housing supplies are insufficient to meet substantial talent demand, and high application thresholds hinder some qualified talents from obtaining effective support. This indicates that further optimization of the policy is required regarding supply-demand balance and precision. It is recommended to increase housing supply and relax application requirements to better meet talent housing needs.

Third, employment support policies have demonstrated a 75% satisfaction rate in recent assessments, indicating their significant effectiveness in facilitating talent employment. For example, Guangzhou’s “Cotton Tree Program” provides returnees with comprehensive support, including employment subsidies and startup funds, effectively alleviating talent employment pressure. However, employment support policies can be further enhanced in employment information services and career development training. Employment information platforms face problems such as untimely information updates and inaccurate job matching, affecting job-seeking efficiency. The vocational training system also needs improvement in aligning training content with market demand and ensuring training quality. Future efforts should focus on optimizing employment information services, accurately pushing employment information, enhancing vocational training quality, and building a more comprehensive employment support system.

Fourth, this section examines entrepreneurship support policies, which have achieved a 70% satisfaction rate. This indicates that these policies have achieved certain results in promoting the entrepreneurial vigor of talents, but there is still room for improvement. Taking Shenzhen’s “Peacock Program” as an example, the policy has achieved some success in implementation. It provides entrepreneurs with capital and venue support, thereby creating a favorable policy environment. However, some entrepreneurs are unaware of the policy details because of insufficient publicity, missing out on opportunities as a result. Additionally, inadequate resource allocation has limited the development of entrepreneurs. These issues highlight the need to strengthen policy promotion so that entrepreneurs can fully utilize the support measures and improve the resource integration platform to help entrepreneurs thrive.

Overall, the Greater Bay Area’s talent policies show relatively strong performance in tax incentives and employment support. However, they require further improvements in housing protection and entrepreneurship support. To improve, policymakers should refine the policy framework according to the changing needs of talents and enhance the policy’s implementation and promotion. This will boost the overall effectiveness of the Greater Bay Area’s talent policies.

#### 5.4.3 Economic and social impact.

The upgrading of the manufacturing sector in the Guangdong-Hong Kong-Macao Greater Bay Area reflects the shift from a “world factory” to an advanced manufacturing hub. Guangdong currently hosts eight industrial clusters valued at over one trillion yuan, including new-generation electronics and information, green petrochemicals, etc. Furthermore, it leads globally in the production of 5G cell phones and air-conditioners, mainly due to Pearl River Delta factories. Additionally, Guangdong has developed ten strategic emerging industry clusters, including semiconductors and integrated circuits, future electronic information, and life and health clusters, thus preliminarily forming a new industrial system of quality productivity. In 2023, the value-added of the manufacturing sector in the Greater Bay Area is expected to exceed US$520 billion, surpassing other global Bay Area regions. A total of 22 companies have been included in the Fortune 500 list, with a combined revenue of approximately US$1.39 trillion. This number surpasses that of the San Francisco Bay Area and is close to that of the New York Bay Area. Brands such as Huawei, Tencent, and BYD have become internationally recognized Chinese business cards, closely linked to the talent policies of Guangdong, Hong Kong, and Macao.

**Fig 11:**
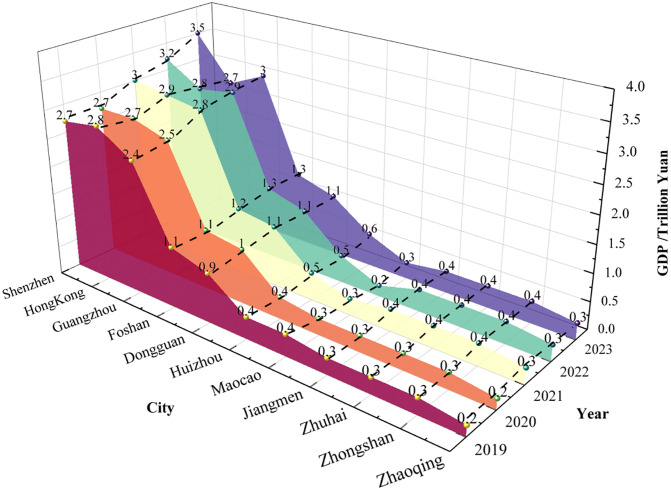
2019-2023 GDP of 11 Cities in Guangdong, Hong Kong and Macao Greater Bay Area.

By the end of 2023, the total economic output of the Guangdong-Hong Kong-Macao Greater Bay Area is anticipated to rise from 10.8 trillion yuan in 2018 to 14 trillion yuan [71]. As depicted in Fig 11, GDP data (in trillions of RMB) for the 11 cities in the Guangdong-Hong Kong-Macao Greater Bay Area from 2019 to 2023 indicate steady economic growth throughout the region. Shenzhen and Guangzhou, the core cities, have witnessed significant average annual growth, with their economies expanding from 2.7 trillion and 2.4 trillion yuan to 3.5 trillion and 3.0 trillion yuan, respectively. This growth is attributed to the innovation-driven upgrading of high-tech industries, financial services, and the manufacturing sector, driven by high-calibre talents. Likewise, Foshan, Dongguan, and Zhaoqing have maintained stable growth. Their GDPs have increased from 1.1 trillion, 0.9 trillion, and 0.2 trillion yuan to 1.3 trillion, 1.1 trillion, and 0.3 trillion yuan, respectively. This progress results from the accelerated transformation of the traditional manufacturing sector into high-end manufacturing, which enhances product value-added and market competitiveness, and represents a new growth highlight for the local economy. Skilled talents have played a pivotal role in this process. However, cities such as Huizhou, Zhuhai, Jiangmen, and Zhongshan, although possessing smaller economies, have also demonstrated steady GDP growth. This reflects the balanced development trend of the Greater Bay Area’s economy, in which the rational flow and effective allocation of talents have played a pivotal role. These data indicate that despite persistent economic disparities among cities in the Greater Bay Area, talent policies have been instrumental in promoting economic growth and balanced social development. Future policies should focus on enhancing regional cooperation, optimizing resource allocation, and promoting synergistic industrial development. This will help reduce inter-city gaps and foster balanced development within the Greater Bay Area. Furthermore, enhancing the precision and effectiveness of talent policies will facilitate a positive interaction between economic growth and social development.

## 6. Conclusions and discussion

### 6.1 Conclusions

This study employs the LDA model to analyze 94 talent policies within the Guangdong-Hong Kong-Macao Greater Bay Area spanning from 2019 to 2023, demonstrating differences in topic intensity and evolutionary trends, informing policy formulation, and addressing the five research questions posed in this paper.

(1) Drawing upon landmark policies, documents, and historical events, along with the guidance of the Outline Plan, the development of the Guangdong-Hong Kong-Macao talent system has been categorized into initial phase (2019), development phase (2020–2022), and deep plowing phase (2023 -present). The primary objectives tackle the challenges of tax synergy, identify high-end and urgently needed talents, and strive to advance the internationalization of the Guangdong-Hong Kong-Macao Greater Bay Area. This progression has encompassed the establishment of the policy framework, the reevaluation of talent qualifications, and the enhancement of implementation details, indicating the ongoing refinement of the policy system.
(2) Keyword theme analysis facilitates policymakers and researchers in grasping the essence of Guangdong-Hong Kong-Macao talent policies. Over the past five years, the themes of talent policies in the Greater Bay Area have encompassed financial talent promotion,Hong Kong and Macao lawyers settlement, high-skill training, skill development, Hong Kong and Macao tax incentives, young talent development, and postdoctoral training grants. Among these, the “Hong Kong-Macao Tax Incentives” serves as the cornerstone, fueling the development of other themes. These tax incentives not only draw talents from Hong Kong and Macao to Guangdong but also spur discussions on practical issues such as system integration and talent mobility.
(3) The evolution of the thematic intensity in the talent policies of Guangdong-Hong Kong-Macao is explored in this study through the utilization of time dimension analysis and heat map visualization. In 2019, the focus of the policies was on the introduction of scientific research and skilled talents. From 2020 to 2022, the focus shifted towards the development of young talents. After 2023, the policies have been tilted towards the service economy, including the settlement of lawyers from Hong Kong and Macao and the promotion of financial talents. This finding corroborates the viewpoints of experts, such as Wang Jianmin from the Development Research Center of the State Council, that the Guangdong-Hong Kong-Macao Greater Bay Area is undergoing a crucial transition from an industrial economy to a service economy [60].
(4) Sankey diagrams are capable of illustrating the flow of talent policy themes, revealing evolutionary relationships, and assisting policymakers in understanding market dynamics. Studies have demonstrated that the talent ecology is intimately linked to the themes of education and training, social security, and scientific and technological innovation. In the future, the Guangdong-Hong Kong-Macao Greater Bay Area aims to deepen integration and establish a talent ecosystem, with talent mobility, as an emerging theme during the development stage, being integrated into international competition and cooperation, thereby expanding the global competitive landscape and striving for the development of a first-class Bay Area.
(5) The talent policy effectiveness assessment shows that the Guangdong-Hong Kong-Macao Greater Bay Area Talent Policy has brought positive results from 2019 to 2023. Talent inflow data comparison reveals a significant rise in talent inflows, proving the policy’s effectiveness in attracting talent. Hong Kong and Macao’s tax incentives have been key in boosting talent concentration and regional appeal. Core cities Shenzhen and Guangzhou achieved economic growth via high-tech and financial services, while Foshan, Dongguan, and Zhaoqing created new growth points through manufacturing upgrades and innovation. Talent policy effectiveness is closely tied to city economic scale and industrial structure. To optimize policies, regional cooperation and resource allocation must be improved to reduce economic gaps and enhance policy effectiveness. These findings offer crucial references for developing more precise talent policies in the Greater Bay Area.

### 6.2 Suggestions

Under the framework of “one country, two systems,” the Guangdong-Hong Kong-Macao Greater Bay Area exhibits both institutional advantages and constraints. To achieve the objective of establishing a high-level talent hub and a world-class Bay Area with international competitiveness, recommendations are proposed based on the development experiences of various international Bay Areas.

#### 6.2.1 Tax incentives:Establishment of an interregional tax cooperation body.

Under the present framework, effective coordination of tax competition in the realm of personal income tax within the Guangdong-Hong Kong-Macao Greater Bay Area, stemming from the three distinct tax systems, poses a challenge for the three regions acting independently. By examining the experiences of the United States, the European Union, and the Beijing-Tianjin-Hebei region, despite the presence of tax competition, these entities have succeeded in fostering orderly competition through the establishment of specialized tax coordination institutions [72]. Illustratively, in 2014, the tax authorities of the Beijing-Tianjin-Hebei region collectively entered into the Beijing-Tianjin-Hebei Collaborative Development Tax Cooperation Framework Agreement, working together to develop and refine a tax coordination mechanism. Subsequent to a series of reforms guided by the agreement, the three regions have achieved mutual recognition of policies, streamlined cross-border business processes, augmented information sharing, advanced cooperation in tax collection and administration, and elevated service standards. Furthermore, the local tax bureaus of Beijing, Tianjin, and Hebei have collaboratively explored innovative approaches to cross-regional tax collection and management, and concluded the Memorandum of Cooperation on Tax Inspection in Beijing, thereby successfully fostering the establishment of a “two-way cross-regional” inspection and coordination mechanism. Retrospectively, it is observed that the Guangdong-Hong Kong-Macao Greater Bay Area has established a construction leading group and corresponding offices. However, in the area of taxation, Guangdong-Hong Kong-Macao have failed to establish an inter-regional planning and management organization for facilitating tax cooperation, and there is a lack of effective tax communication and coordination mechanisms. This has resulted in the slow progress of tax cooperation and reduced efficiency of such cooperation. Therefore, it is suggested that referencing be done to domestic and overseas experience, and that personal income tax be utilized as a pilot project. Based on the Outline Plan and framework agreements such as the Hong Kong-Macao Closer Economic Partnership Arrangement (CEPA), the State Administration of Taxation should initiate the establishment of a tax cooperation organization for personal income tax, with the Guangdong Provincial Taxation Bureau taking a leading role in collaborating with the tax authorities of Hong Kong and Macao. The goal is to facilitate the establishment of a unified system of tax collection and administration among Guangdong-Hong Kong-Macao.

#### 6.2.2 Talent Ecology:Implementation of the talent synergy development strategy.

The successful experiences of the San Francisco Bay Area demonstrate that education, science, and technology, along with talent, possess a mutually supportive, dialectical, and unifying intrinsic linkage, thereby enabling the maximization of the synergistic effect of “1 + 1+1>3” solely through synergistic development [73]. Livelihood protection serves as the “ballast stone” of social stability, acting as the “peace of mind pill” for talents working in other locations. By implementing measures such as the mutual recognition of social security qualifications for scientific and technological talents and ensuring the protection of medical care and children’s education, the Japanese government has substantially increased the attractiveness of the Bay Area to international talents [74]. Essentially, the synergistic development of education, science, technology, talent, and social security constitutes the key to achieving the integration of talent ecology and the construction of an international first-class Bay Area. Given this, the Guangdong-Hong Kong-Macao Greater Bay Area should draw upon these successful experiences and adopt a diversified strategy for the synergistic development of talents: (1) Education: The potential universities in the Bay Area should be fully supported to aspire towards world-class status, establishing a high-quality local tertiary education system, and forming high-level university clusters and tertiary education hubs, thereby attracting global talents to converge in the Greater Bay Area and fostering high-caliber talents therein; (2) Science and technology: The universities located in the Guangdong-Hong Kong-Macao Greater Bay Area should be fully leveraged to fulfill their role in fundamental scientific research, aligning with the national strategic and economic priorities. Concurrently, firms should be incentivized to emulate Silicon Valley in cultivating and driving technological innovations, as well as undertaking pioneering research and development. For this purpose, firms must be encouraged to adhere to the principle of “mutual benefit, shared risk, complementary advantages, and cooperation”, enhance their cooperation with academic and research institutions, collaborate to overcome technological challenges, and cultivate industry-academia-research collaborative enterprises. (3) Talents: Leverage the unique advantages of “one country, two systems, three tariff zones, and three currencies” to foster an open and inclusive cultural atmosphere, and strengthen the integration of the regulatory system to facilitate the free flow and optimal allocation of talents. Furthermore, all stakeholders should collectively strive to continuously refine the mechanism for nurturing, attracting, and utilizing talents, aiming to position the region as a premier global talent and innovation hub. (4) Social security: The Guangdong-Hong Kong-Macao Greater Bay Area should establish high-end international talent communities in various locations, investigate talent protection services that meet international standards, and enhance the supporting facilities, such as international schools and healthcare institutions, thereby addressing the concerns of international talents.

#### 6.2.3 Talent Mobility: Establishment of a mechanism for mutual recognition of top talents.

The ease of immigration policies serves as the “valve” for the free flow of talent. Within the European Union, the implementation of the principle of residence-based citizenship has significantly reduced the emphasis on national borders, enabling individuals holding passports from any European Union member state to reside, work, study, and practice freely and without restriction within the Union. In contrast, facing similar challenges is the Guangdong-Hong Kong-Macao Greater Bay Area, characterized by its “one country, two systems,” three legal systems, and a separate customs territory. Adopting the “European Union citizenship” model directly may pose immediate implementation pressures and challenges on Hong Kong and Macao [75]. Therefore, priority should be given to high-end talent groups in the Guangdong-Hong Kong-Macao Greater Bay Area, with immigration and residence preferences granted based on residency, rather than household registration. Additionally, to accelerate the harmonization of talent mobility and shared services, the Greater Bay Area should establish a unified service standard and information technology platform tailored for high-end talents, integrate service programs, and set unified benchmarks. Consequently, a robust service network and on-site service system ought to be established, while avenues for cooperation, such as remote personnel agency, talent dispatch, and human resources outsourcing, ought to be actively expanded to facilitate rapid talent mobility. Furthermore, addressing the key issue of mutual recognition of vocational qualifications and eliminating barriers to occupational mobility should be a priority for the Guangdong-Hong Kong-Macao Greater Bay Area. By drawing upon the successful experience of the European Union’s vocational qualification system and the mutual recognition of vocational qualifications between the Guangdong Pilot Free Trade Zone and Hong Kong and Macao, a set of reference standards and a conversion mechanism for the mutual recognition of skilled personnel qualifications should be established, in collaboration with government agencies and professional organizations in the three regions. Consequently, the mutual recognition system for the practicing qualifications of talents in the Guangdong-Hong Kong-Macao Greater Bay Area, particularly in key sectors such as law, finance, education, healthcare, and accounting, will be substantially promoted, thereby advancing the integration of talents in Guangdong-Hong Kong-Macao to a higher level of development.

#### 6.2.4 Addressing challenges in talent policy implementation and evaluation systems.

In the implementation of talent policies in the Guangdong-Hong Kong-Macao Greater Bay Area, although many policies have been introduced to attract global researchers, their sustained implementation has been less than ideal. The arbitrary suspension of some policies has not only weakened the long-term effectiveness of talent attraction and retention but also undermined the credibility of the policies. To address this issue, it is imperative to establish a more stable and forward-looking policy framework. Specifically, first, it is necessary to clarify policy objectives and establish a clear timeline to ensure consistency and predictability of policies; second, regular evaluations of policy implementation outcomes should be conducted to promptly identify issues and make corresponding adjustments; additionally, stakeholders, including researchers and industry representatives, should be actively involved in the policy formulation and evaluation processes. This not only enhances the targeting and adaptability of policies but also improves their sustainability. Meanwhile, China’s existing peer review system still has certain limitations in identifying individuals with significant achievements in various fields, which may lead to some high-quality talent being overlooked. To optimize this system, the following measures are recommended: first, expand the dimensions of evaluation criteria. In addition to traditional metrics such as citation counts, more diversified standards should be incorporated to comprehensively recognize different types of talent and their achievements; second, strengthen international cooperation by inviting international experts to participate in evaluations, introducing global standards and perspectives to enhance the internationalization of evaluations; third, enhance the transparency of the evaluation process and establish corresponding accountability mechanisms to ensure the fairness and objectivity of evaluations. Additionally, while some talents currently enjoy high incomes, their output efficiency remains relatively low, which to some extent reflects unreasonable aspects of the talent incentive mechanism, thereby impacting overall efficiency. To address this issue, the following strategies can be adopted: First, establish a performance-based incentive mechanism, closely linking incentive measures to actual performance, ensuring that high income aligns with high output, and fully stimulating the creativity and initiative of talents; Second, establish a continuous monitoring and evaluation system to regularly track and comprehensively assess talent performance, promptly identify issues, and provide feedback and support to help talent continuously improve their capabilities; Third, vigorously cultivate an innovative culture, create a positive atmosphere that encourages innovation and continuous learning, and motivate talent to produce more high-quality outcomes, providing a solid talent foundation for the development of the Greater Bay Area.

### 6.3 Research shortcomings and prospects

This study faced specific limitations during data collection and analysis. Although the sample size was expanded through diverse channels, potential data bias may still exist. For example, data incompleteness may result from certain policy documents being unavailable to the public or difficult to access. Furthermore, the LDA model has limitations in the precision of topic extraction and semantic comprehension, which may lead to an incomplete capture of the subtleties and deeper implications within policy texts. Future research can improve data quality by expanding data sources and further optimizing data collection methods. Additionally, combining other text analysis methods, such as TF-IDF, with the LDA model can supplement the LDA model, thereby improving the accuracy and comprehensiveness of topic mining. Regarding research directions, it is advisable to deeply track the long-term impact of talent policies and assess their sustainability. Comparative studies with other emerging economic regions should also be conducted to provide a broader international perspective for optimizing talent policies in the Greater Bay Area. These initiatives will help to further enhance the effectiveness of talent policies in the Guangdong-Hong Kong-Macao Greater Bay Area and strengthen the region’s global competitiveness.

## Supporting information

S1 File Policy Document (English Version). (RAR)
